# Psychometric evaluation of personal evaluation of transitions in treatment amongst patients with schizophrenia

**DOI:** 10.4102/sajpsychiatry.v27i0.1491

**Published:** 2021-03-05

**Authors:** Oladipo A. Sowunmi, Peter O. Onifade, Oluwatoyin R. Sowunmi

**Affiliations:** 1 Department of Clinical Services, Faculty of Psychiatry, Emergency Psychiatric Unit, Neuropsychiatric Hospital Aro, Abeokuta, Nigeria

**Keywords:** adherence, schizophrenia, PETiT, validation, Nigeria, Aro

## Abstract

**Background:**

Monitoring the antipsychotic medication adherence of outpatients with schizophrenia has focused majorly on the medication construct of adherence, whilst neglecting its psychosocial construct.

**Aim:**

The aim of this study was to provide the psychometric properties of personal evaluation of transitions in treatment (PETiT).

**Setting:**

This study was conducted at the Neuropsychiatric Hospital, Aro, Abeokuta, Ogun State, Nigeria.

**Methods:**

This is a study of diagnostic accuracy of patients with a diagnosis of schizophrenia. Calculation of the sample size and oversampling was calculated as proposed by Cochrane.

**Results:**

The Cronbach’s *α* in this study at baseline was 0.82. Intra-class correlation coefficient was 0.81 (*p* ≤ 0.001). Medication construct was loaded into two factors or components whilst the psychosocial construct was loaded into four factors.

**Conclusion:**

The data depicted here indicated a successful validation and presentation of psychometric properties of PETiT which is self-administered, user friendly, psychometrically sound and sensitive to changes associated with treatment over time.

## Introduction

In the management of patients with schizophrenia, one of the main targets after diagnosis is treatment which may include physical, psychological or social interventions.^1,2,3,4,5^ To achieve effective and efficient treatment in this group of patients, the use of antipsychotic medication cannot be overemphasised as a previous study had reported that non-adherence could be as high as 89%.^6,7,8^ Furthermore, in order to ensure optimal progress in treatment, medication given would have to be monitored.

Monitoring the antipsychotic medication adherence of outpatients with schizophrenia has remained a major challenge because mental health practitioners usually rely on patients’ self-report of adherence. Although various methods have been advocated none is without its own particular shortcoming.^2,3,4,5,9^ Whilst the need of an effective adherence assessment tool in schizophrenia has been hindered by a deficit in an objective, valid and reliable assessment methods capable of serving as a gold standard for evaluating antipsychotic drug use within the community in Nigeria, it remains pertinent that tools should be available with proper validation and psychometric properties so that results can be compared and solutions proposed can be implemented generally amongst patients with schizophrenia.^2,3,4,5,9^

Be that as it may, the outcome or solution proposed need to have adequately considered the proximal and distal side treatment outcome as proposed by Voruganti and Awad.^10^ The proximal outcome was opined to include illness component that was likely to change with the commencement of antipsychotic medication whilst distal outcome is made up of the cumulative effect of treatment in addition to extrinsic factors. Thus changes in symptoms and side effects of medication are proximal outcomes whilst functional status and quality of life are distal outcomes.^3^ However, when treatment outcome becomes evident, it is difficult to separate the contributions of various confounders, thus the question remains: are the changes because of either the combination of therapeutic factors and/or non-therapeutic factors or either of them. To resolve these issues, it seems important to identify the dynamics of transition between proximal and distal outcomes by using evaluation tools which are equipped to do so. One of these instruments is the personal evaluation of transitions in treatment (PETiT) which has been reported to provide the missing link between antipsychotic drug therapy, and problems with medication adherence, clinical instability, compromised quality of life and increased resource utilisation.

Most of the earlier tools used in Nigeria have only evaluated the proximal side of adherence, none has used an instrument that evaluated both the proximal and distal outcome of antipsychotic treatment inpatient suffering from schizophrenia. Although the use of self-administered questionnaires has remained common in Nigeria, previous studies^11,12,13^ performed in Nigeria have looked at medication adherence in the proximal end of the outcome without reporting the psychometric properties of the evaluation tool they used. In this study, our aim is to determine the psychometric properties of PETiT amongst outpatients with schizophrenia at the Neuropsychiatric Hospital, Aro, Abeokuta, Ogun State, which will evaluate both the proximal and distal outcome of antipsychotic treatment.

## Methodology

### Study site

The study was carried out at the outpatient clinic of the Neuropsychiatric Hospital, Aro, Abeokuta, Ogun State. The hospital initially started at its annex in 1944 as an asylum for soldiers who were repatriated home because of mental illness after the Second World War. The current main site, Aro was built in 1954 when the need for a modern psychiatric hospital arose. The hospital renders services to patients from all over Nigeria and from the neighboring West African countries. It has a total capacity of 546 beds for inpatient care, 153 beds at the main hospital and 393 beds at the Lantoro annex.^6^

All new patients are seen at the Assessment or Emergency unit of the hospital from where they are transferred to either the out-patient clinic or the wards. Those discharged from the wards are also subsequently followed-up at the out-patient clinic. No new patient is seen at the out-patient clinic of the hospital. The clinic runs on Mondays, Tuesdays, Thursdays and Fridays, and a Senior Registrar’s clinic on Wednesdays.^6^ From the statistics at the Medical Records Department of the hospital, about 130 patients are seen daily at the out-patient clinic. Of these, 41.9% are patients with schizophrenia, about 12% are patients with bipolar affective disorder and 3.2% are patients with substance use disorder.^6^

### Sample size determination

Calculation of the sample size and oversampling was calculated as proposed by Cochrane.^14^ The calculation gave a total sample size (*n*) of 220.

### The instrument

Personal evaluation of transitions in treatment was originally designed for use in acute or persistently psychotic disorders requiring or already receiving antipsychotic drug treatment as a structured, self-report evaluation tool containing a total of 69 items, with four items in each of the 12 domains. Subsequently, using factor analysis and other standard statistical techniques, a 30-item questionnaire without subscales (Symptom subscale, Functional Status subscale and Standard of Living subscale) was produced. Items from the previous domains were presented randomly, often reversing the wordings of the sentences. It has an introductory aspect which outlines the purpose of the questionnaire and the need to consider their health in the last 1 week and a choice of one of three response (‘often’, ‘sometimes’ or ‘never’).^4,10,15^

These responses represent the frequency of experiencing a specific item during the period of 1 week and the answer assigned a rating of 2, 1 and 0. A score of 2 represents a positive change, and ‘0’ a change in the negative direction, which is subject to the way the words in the items are framed. Total scores are derived from the summation of the individual items and the authors ensured that attention was given to exclude any cultural and health-services related items, so that the scale could be used in a variety of health care facilities.^4,10,15^

The total number of participants used in the initial study by Voruganti and Awad^10^ was 320 and most of them did not perceive the instrument as difficult with mean scores on the visual analogue scale as follows: clarity (8.2 ± 1.2), self-rated accuracy of response (8.0 ± 1.8) and cognitive burden (1.8 ± 0.9). Age, gender, educational level and marital status were not observed to be confounding factors with PETiT. The scale assesses two highly relevant domains for schizophrenia: adherence-related attitude (includes six items reflecting adherence and feelings towards medication) and psychosocial functioning (24 items describing patient characteristics such as clarity, energy, concentration, functioning, sex drive and memory).^4,10,15^

Psychosocial functioning can be assessed further within four subdomains: social functioning (four items on trust, confidence and interactions), activity (seven items reflecting energy and ability to conduct daily tasks), cognitive (seven items on clarity, concentration and communication) and dysphoria (six items on happiness, future and self-esteem). The PETiT total score ranges from 0 to 60, with higher scores denoting better patient health-related quality of life (HRQoL). The scale has high internal consistency (Cronbach’s *α* = 0.92) and high test-retest reliability (0.97; *p* < 0.001).^4,10,15^ The 30-item PETiT can be found in Table 1.

**TABLE 1:** Mean scale score (at T^1^ and T^2^) and frequency of item scale of respondents at T^1^.

Question		Often		Sometimes		Never	
		*n*	%	*n*	%	*n*	%
General questions							
1	My mind is sharp and clear	114	51.9	76	34.5	30	13.6
2	I am worried about what is happening to my health	51	23.2	108	49.1	61	27.7
3	I feel dull and sluggish	42	19.1	93	42.3	85	38.6
4	I believe that people feel comfortable around me	137	62.3	56	25.4	27	12.3
5	I feel too tired to do things that I should do	45	20.5	100	45.5	75	34.0
6	I find it hard to come up with new ideas	44	20.0	75	34.1	101	45.9
7	I am unable to trust people	47	21.4	97	44.1	76	34.5
8	I am satisfied with my life	78	35.5	90	40.9	52	23.6
9	I am able to concentrate on reading or television	121	55.0	73	33.2	26	11.8
10	I am unhappy	38	17.3	91	41.3	91	41.4
11	I have family or friends who really understand me	146	66.4	49	22.3	25	11.3
12	My sex drive is weak	40	18.2	59	26.8	121	55.0
13	I am able to communicate better with people	137	62.3	59	26.8	24	10.9
14	Chores such as cleaning, washing and shopping are too much for me	36	16.4	85	38.6	99	45.0
15	I am able to remember things easily	111	50.5	88	40.0	21	9.5
16	I feel ready to work either as a volunteer or for pay	122	55.5	68	30.9	30	13.6
17	I feel good about myself	120	54.5	79	36.0	21	9.5
18	My future seems gloomy	89	40.4	62	28.2	69	31.4
19	I avoid meeting new people	38	17.3	63	28.6	119	54.1
20	I feel weird and strange	26	11.8	73	33.2	121	55.0
21	I can handle the daily hassles of life	97	44.1	83	37.7	40	18.2
22	I dislike the way I look	28	12.8	63	28.6	129	58.6
23	I am not sleeping well	56	25.5	76	34.5	88	40.0
24	I am able to do things as well as other people	124	56.4	67	30.5	29	13.1
**Questions about medication**							
25	I forget to take my medication	21	9.5	56	25.5	143	65.0
26	My medication is helping me	170	77.3	35	15.9	15	6.8
27	I dislike my current medication	39	17.7	53	24.1	128	58.2
28	Friends and family believe that my current medication is good for me	160	72.7	43	19.6	17	7.7
29	Taking medication is unpleasant	35	15.9	63	28.6	122	55.5
30	I feel that the good things about taking medication outweigh the bad	131	59.6	52	23.6	37	16.8

Personal evaluation of transitions in treatment (PETiT) total score: Mean (SD)T^1^ = 40.58 ± 8.62; Mean (SD)T^2^ = 49.83 ± 8.62.

### Study design

This is a study to determine the psychometric properties of PETiT. Respondents were recruited using a systematic random sampling of every fourth patient registered to be seen at the outpatient clinic of the hospital. Respondents with a diagnosis of schizophrenia, aged 18 years and above, and who were able to read and write in English were recruited. The study spanned the period June 2016 to June 2017. The retest was conducted after 1 year (over 4–8 weeks) of the initial test because of the difference in the duration of clinic visit given in the different unit of the hospital.

### Data analysis

Data were analysed using the Statistical Package for Social Science (SPSS version 23) computer software. The level of significance was set at *p* ≤ 0.05. Intra-class correlation coefficient (ICC) was used to determine the internal consistency of the instrument using responses from the first test and Cronbach’s alpha for the entire scale. Intra-class correlation coefficient was also used to determine the test-retest reliability of the instrument using total scores at test and retest. Factor analysis using principal component analysis with Varimax rotation was used to confirm the robustness of the original construct. In order to confirm the robustness of the original construct, the items were split into two (medication domain and psychosocial domain) and extraction was done based on fixed number of factors (which is four) as was done by the original author.^5,10^ An item-total correlation was also done with ICC coefficient using two-way mixed method with absolute agreement to determine the concurrent validity of the questionnaire.

### Ethical consideration

Ethical approval was obtained from the research and ethics committee of the Neuropsychiatric Hospital, Aro, Abeokuta, Ogun State, Nigeria and permission was obtained from the managing consultants. Consent was obtained from all participants and attention of the managing consultants was drawn to the corresponding patients who had problems with medication adherence. Ethical clearance number was NHREC/24/07/2013.

## Result

Table 2 shows the socio-demographic variables of respondents. The mean age of participants in this study was 38.40 ± 13.47. The rest of the socio-demographic variables are depicted on Table 2. Table 1 shows the frequency of response of each of the items of the PETiT questionnaire. The mean total scale score of respondents in this study was 40.58 ± 8.62. Other details are highlighted on Table 1.

**TABLE 2:** Sociodemographic variables of respondents.

Variables	Descriptive Statistics			
	*n*	%	Mean	±SD
Age	-	-	38.40	±13.47
**Gender**				
Male	99	45.0	-	-
Female	121	55.0	-	-
**Marital status**				
With a partner	96	43.6	-	-
Without a partner	124	56.4	-	-
**Level of education**				
No formal or primary education	55	25.0	-	-
Secondary education	89	40.5	-	-
Tertiary education	76	34.5	-	-
**Ethnicity**				
Yoruba	199	90.5	-	-
Other tribe	21	9.5	-	-
**Religion**				
Christianity	163	74.1	-	-
Islam or traditional African religion	57	25.9	-	-

The questionnaire was administered to 220 respondents with a mean duration of 5 min (range 3–12 min) for completion. Patients with acute and severe psychotic symptoms were unable to participate because of poor concentration, aggression, restlessness and irritability. The Cronbach’s *α* in this study at Test 1 (T^1^) was 0.82 (Cronbach’s *α* if item deleted was not significant for any of the 30-item analysed) with an ICC of 0.81 and a *p* value of < 0.001. The Cronbach’s *α* for the test-retest was 0.22 with an ICC of 0.16 and a *p* value of 0.03. Other details can be found on Table 3.

**TABLE 3a:** Cronbach’s alpha and intra-class correlation coefficient of personal evaluation of transitions in treatment.

Intra-class correlation†		95% confidence interval		*F* test with true value 0			
		Lower bound	Upper bound	Value	*df*1	*df*2	*p*
**Intra-class correlation coefficient**							
Single measures	0.12‡	0.090	0.150	5.430	219	6351	< 0.001
Average measures	0.81§	0.767	0.841	5.433	219	6351	< 0.001
**Reliability statistics (at initial test)**							
Cronbach’s *α*	0.82	-	-	-	-	-	-

Two-way mixed effects model where people effects are random and measures effects are fixed.

† , Type A intra-class correlation coefficients using an absolute agreement definition.

‡ , The estimator is the same, whether the interaction effect is present or not.

§ , This estimate is computed assuming the interaction effect is absent, because it is not estimable otherwise.

**TABLE 3b:** Cronbach’s alpha and intra-class correlation coefficient of personal evaluation of transitions in treatment.

Intra-class correlation†		95% confidence interval		*F* test with true value 0			
		Lower bound	Upper bound	Value	*df*1	*df*2	Sig
**Intra-class correlation coefficient**							
Single measures	0.09‡	−0.03	0.21	1.287	219	219	0.03
Average measures	0.16§	−0.06	0.34	1.287	219	219	**0.03**
**Reliability statistics (test versus retest)**							
Cronbach’s *α*	0.22	-	-	-	-	-	-

Two-way mixed effects model where people effects are random and measures effects are fixed.

† , Type A intra-class correlation coefficients using an absolute agreement definition.

‡ , The estimator is the same, whether the interaction effect is present or not.

§ , This estimate is computed assuming the interaction effect is absent because it is not estimable otherwise.

As mentioned earlier, the factor analysis was done in two stages: medication construct and psychosocial construct. Medication construct loaded into two factors or components which were as follows: factor 1 was made up of questions 25, 27 and 29 whilst factor 2 was made up of questions 26, 28 and 30. The psychosocial construct loaded into four factors and are as follows: factor 1 was made up of questions 6, 7, 8, 10, 14, 19, 20 22 and 23; factor 2 was made up of questions 4, 9, 11, 12, 13, 15 17 and 18; factor 3 was made up of questions 1, 16, 21 and 24 whilst factor 4 was made up of questions 2, 3 and 5. Other detail can be seen on Table 4.

**TABLE 4:** Rotated component matrix of 30-item personal evaluation of transitions in treatment.

Questions	Component			
	1	2	3	4
**Rotated component matrix‡ (psychosocial domain)**				
PETiT General Question Test Q7	0.614	−0.103	0.078	−0.049
PETiT General Question Test Q22	0.602	0.121	−0.042	0.135
PETiT General Question Test Q14	0.596	0.172	0.012	0.105
PETiT General Question Test Q20	0.582	0.051	0.000	0.217
PETiT General Question Test Q10	0.547	0.067	0.183	0.231
PETiT General Question Test Q6	0.537	0.063	0.222	0.373
PETiT General Question Test Q19	0.517	−0.061	−0.021	0.018
PETiT General Question Test Q23	0.443	0.000	0.257	−0.143
PETiT General Question Test Q12	0.378	0.206	−0.284	0.151
PETiT General Question Test Q13	0.031	0.689	0.099	0.074
PETiT General Question Test Q11	0.164	0.669	0.012	−0.059
PETiT General Question Test Q15	−0.102	0.567	0.227	0.213
PETiT General Question Test Q9	0.005	0.567	0.210	0.064
PETiT General Question Test Q4	0.044	0.554	0.021	−0.100
PETiT General Question Test Q17	−0.069	0.454	0.452	−0.077
PETiT General Question Test Q21	0.082	0.025	0.756	−0.084
PETiT General Question Test Q16	0.089	0.112	0.569	0.110
PETiT General Question Test Q24	0.095	0.332	0.538	0.009
PETiT General Question Test Q1	−0.022	0.355	0.527	0.094
PETiT General Question Test Q8	0.047	0.397	0.400	0.157
PETiT General Question Test Q2	0.002	0.036	−0.134	0.730
PETiT General Question Test Q3	0.242	0.086	0.227	0.718
PETiT General Question Test Q5	0.388	−0.053	0.114	0.577
PETiT General Question Test Q18	0.221	0.002	−0.196	0.241
**Rotated component matrix† (medication domain)**				
PETiT Medication Test1q25	0.785	−0.102	-	-
PETiT Medication Test1q29	0.770	0.069	-	-
PETiT Medication Test1q27	0.768	0.262	-	-
PETiT Medication Test1q26	0.132	0.749	-	-
PETiT Medication Test1q28	0.143	0.733	-	-
PETiT Medication Test1q30	−0.094	0.731	-	-

PETiT, personal evaluation of transitions in treatment.

Extraction method: Principal component analysis. Rotation method: Varimax with Kaiser normalisation.

† , Rotation converged in seven iterations;

‡ , Rotation converged in 3 iterations.

## Discussion

The personal evaluation of transition in treatment was designed to explicitly monitor subjective changes in adherence attitude and psychosocial functioning in the context of antipsychotic drug therapy in patients diagnosed with schizophrenia. Overall, we observed that PETiT had valid and reliable psychometric properties when compared to those performed outside of Nigeria. The Cronbach’s alpha and ICC were also comparable to those outside Nigeria and subjective assessment of changes in adherence attitude and psychosocial functioning in the context of antipsychotic drug therapy in patients diagnosed with schizophrenia showed improvement over 1 year as reported by the respondents.

The mean scores at baseline and at 1 year (40.58 ± 8.62; 49.83 ± 8.62) observed in this study was higher than that reported in a previous study^15^ conducted in the United States of America. However, the relationship between the baseline score and the score after a year in this study was not statistically significant. An explanation is that the study conducted in America was drug-specific, which was not the case in our own study. The evaluation in our study included patients taking both typical and atypical antipsychotics without delineation into typical and atypical or to specific antipsychotic medications which was the sub-classification conducted in the study by Awad and his coworker before comparison was done between baseline and 1 year.

The Cronbach’s alpha in this study was moderate to high which is similar to what was achieved by the initial authors whilst validating the 30-item instrument.^10^ It appears that the idea of using PETiT, as an instrument to facilitate the formulation and validation of the concept of intermediate outcomes in the context of antipsychotic drug therapy, was justified in our study. This shows that the ideological approach used by the authors to capture the concerns and expectations of patients was adequate. Moreover, just as was done in the initial study, the instrument was subdivided into two groups (medication construct and psychosocial construct) for the factor analysis. In our study, the medication construct appears to have loaded into two components: component one was made up of questions 25, 27 and 29 addressing the patients’ attitude to antipsychotic medication and the second component was made up of questions 26, 28 and 30 addressing the patients’ subjective believe of the effect of antipsychotic medication. Although questions 25 through to 30 address medication adherences, it appears that patients within our clime tend to view them as two different constructs.

Furthermore, as regards the psychosocial construct, the 26-items loaded four factors. Factor 1 was made up of questions 6, 7, 8, 10, 14, 19, 20 22 and 23 which is in keeping with the cognitive scale (which evaluated clarity, concentration and communication) of the initial authors and both cases were made up of seven items. The second factor was made up of questions 4, 9, 11, 12, 13, 15 17 and 18 appeared to be like the social functioning subscale, which measured trust, confidence and interaction. However, unlike the initial validation, our subscale was made up of eight items rather than the four-item subscale of the initial authors. This, in our assessment, appears to be related to cultural interpretations of some items which may account for the observed loading in factor 2. Factor 3 was populated with questions 1, 16, 21 and 24 which are keeping with the subscale on activity. None the less, our own subscale was made up of four items, whilst the original subscale was made up of seven items. In addition, the fourth factor, made up of questions 2, 3 and 5 is akin to the dysphoric subscale, was made up of three items rather than the six items found in the original validation.

## Conclusion

These results were critical in establishing the subjective dimension of patients as the missing link between antipsychotic drug treatment, problems with treatment adherence, clinical stability and increased resource utilisation. In conclusion, the data depicted here indicated a successful validation and presentation of psychometric properties of PETiT which is self-administered, user friendly, psychometrically sound and sensitive to changes associated with treatment over time. To the best of our knowledge, this is the first time this instrument has been validated in Nigeria and in Africa. This study provides a standardised instrument for the evaluation of psychosocial issues in antipsychotic treatment in patient diagnosed with schizophrenia. This is likely to impact psychosocial interventions such as psychoeducation, family therapy and cognitive behavioural therapy to mention a few. Furthermore, it could complement the subject evaluation given by the nursing staff of patients diagnosed with schizophrenia during ward rounds. This might serve as objective complement to subject reports of patients with schizophrenia.

However, this study is not without its limitations. This study was only conducted in a single psychiatric setting in Nigeria and although it serves patients all over the country and its adjourning countries like the Republic of Benin and Cameroon. The report should be taken with caution in terms of generalisation to other countries in West Africa or Africa at large. Future studies should evaluate improvements based on specific medication used at baseline as this is likely to produce an optimal result. In addition, future studies should evaluate the correlation of subject reports of nursing contribution to the evaluation of improvement of patients on antipsychotic drug therapy with a diagnosis of schizophrenia. This will determine if this instrument could be a reliable guide during ward rounds and thus help in policy formation and hospital legislation. Finally, it is hoped that this instrument can be pushed as a tool that will incorporate client-centred measure in our quality assurance packages.
